# New products

**DOI:** 10.1155/S1463924699000176

**Published:** 1999


Journal of Automated Methods & Management in Chemistry, Vol. 21, No. 4 (July–August 1999) pp. 139–143

### New products

### CD-ROM guides for ELAN 6000 sampling accessories

Perkin-Elmer Sciex and CETAC Technologies are producing a series of interactive CD-ROMs describing the installation, set-up, optimization and typical applications of CETAC's main sampling accessories, used with the ELAN 6000 inductively coupled plasma mass spectrometer (ICP-MS).

The ELAN 6000 is a fully automated, computer-controlled instrument, designed for both routine and research trace element determination in environmental, clinical, geochemical, semiconductor, nuclear, food and metallurgical application areas.

The following CDs are available: 'Using the MCN-6000 Desolvating Microconcentric Nebulizer with the ELAN 6000 ICP-MS' and 'Using the LSX-200 Laser Ablation System with the ELAN 6000 ICP-MS'.

*For further information contact Perkin-Elmer Limited, Post Office Lane, Beaconsfield, Bucks HP9 1QA, UK. Tel: +44 (0)1494 676161; Fax: +44 (0)1494 679331/3; e-mail: greenca@eur.perkin-elmer.com;* Internet *http://www.perkin-elmer.com*

### New 4100 Series from Shimadzu for high-performance on-line water quality analysis

The 4100 Series is a new range of total organic carbon (TOC) on-line water quality analysers from Shimadzu which will be shown for the first time at this year's WET Show (Stand number: B6).

The Series comprises the TOC-4100 for TOC measurement, the TN-4100 for TN (total nitrogen) measurement, and the TOCN-4100 for both TOC and TN measurement. All analysers provide quick and accurate analysis of organic carbon and nitrogen in aqueous matrices, and are therefore ideal for use in applications, e.g. the management of influent and effluent for waste water treatment, management of plant waters, monitoring of boiler and condensate water, monitoring of drinking and highly processed water as well as natural water, and monitoring of water quality for regulatory reporting.

The TOC-4100 is a high-performance analyser which adopts the same 680 °C catalyst-aided combustion technique as the successful TOC-5000(A) Series of instruments, which are used worldwide in a variety of demanding applications.

The analyser allows any of three TOC measurement modes to be selected, depending on sample characteristics—TOC (NPOC, non-purgeable organic carbon) measurement using the acidify/sparge process, TOC measurement via differential total carbon (TC) and IC (inorganic carbon) components, and TOC measurement by addition of POC (purgeable organic carbon) and NPOC. Total nitrogen measurement can also be added.

A complete line of options is also available, to accommodate samples with suspended particles, slime or seaweed.

The TN-4100 uses a combustion/chemiluminescence detection technique. It features an automatic dilution function as standard which enables TN measurement of concentrations up to 4000 ppm, and a short measurement cycle of 4 min, which means that sudden changes in TN measurement can be captured and more effective monitoring is possible by switching between multiple streams. Measurement of sea water can also be accommodated by the system.

The TOCN-4100 enables simultaneous measurement of TOC and TN with a single instrument. A compact water analyser, the TOCN-4100 is unique in its ability to handle TOC analysis by combustion oxidation/infrared detection and TN analysis by combustion/chemiluminescence, all in a single unit.

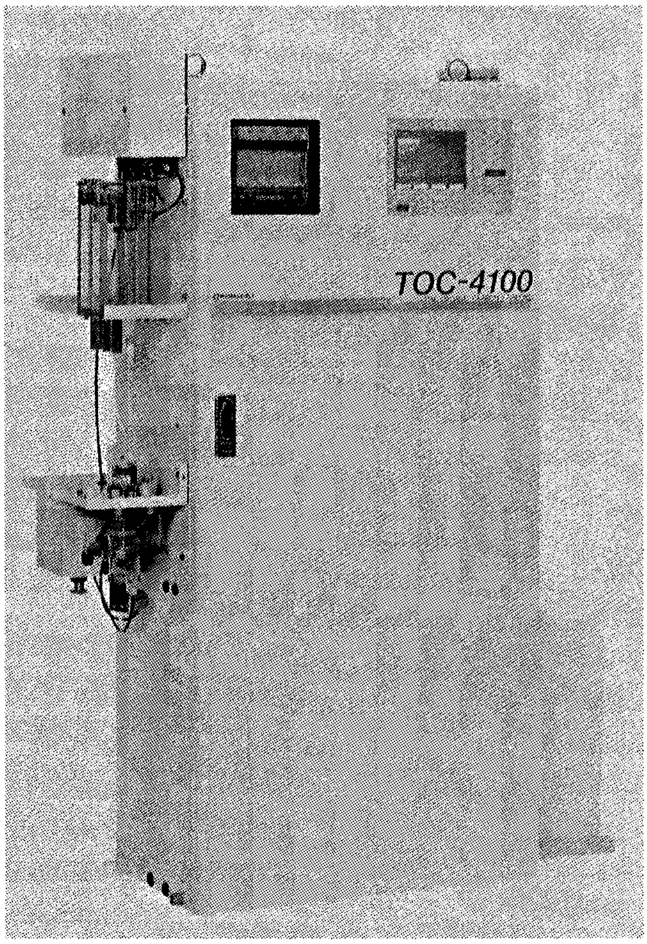

TOC and TN are simultaneously measured with results available in as little as 4 min. The unit also features automatic dilution, calibration and various input/output functions as standard, along with a range of options, including a suspended particle flow line switcher, which facilitates its use in a wider variety of applications.

'A great deal of thought has gone into the development of this new Series of Shimadzu instruments, to give users a raft of new features—many of them unique to our technology', said Peter Sidhu of Shimadzu.

'The range of applications in which on-line TOC technology can be used continues to expand, while TN measurement is gaining in importance since nitrogen is considered one of the factors which contribute to the over-proliferation of organisms in enclosed bodies of water so the management of public water sources and treatment plants demands the exact monitoring of nitrogen levels'.

'We are very proud of these new analysers, all of which use proven combustion technology to truly lead the field in on-line water quality analysis'.

*Free literature on the new 4100 Series is available from Shimadzu at Shimadzu Europa (UK Branch), Mill Court, Featherstone Road, Wolverton Mill South, Milton Keynes MK12 5RE, UK. Tel: +44 (0)1908 552200; Fax: +44 (0)1908 552211; e-mail: Sales@Shimadzu.co.uk*

### Free spectorscopy software catalogue

*Galactic releases new product catalogue for GRAMS/32 and its add-on applications*

Salem, NH—10 December 1998: This new catalogue features Galactic's innovative line of spectroscopy software products. The catalogue includes detailed product descriptions and graphics on GRAMS/32, the complete data processing and data management package; Spectral ID, the spectral library searching tool that supports all the popular commercial libraries; PLSplus/IQ, the spectroscopy chemometrics toolbox; and GRAMS/3D, the real-time 3D visualization package. System requirement details are also given for each product. For a copy of this 12 page, coloured product catalogue, contact Galactic.

Galactic Industries Corporation is the leading developer of spectroscopy software for PCs. Galactic products are designed to integrate all laboratory data in one common software package for data processing, viewing and managing. Galactic is headquartered in New Hampshire at 395 Main Street, Salem, NH 03079. Tel: +1 (603) 898-7600. World Wide Web http://www. galactic.com.

*For more information contact: Christine Cassa, Marketing Communications Coordinator. Tel: +1 603 898 7600; clc@galactic.com*

### Electronic Handbook of Infrared and Raman Spectra of Inorganic Compounds and Organic Salts

The *Electronic Handbook of Infrared and Raman Spectra of Inorganic Compounds and Organic Salts* provides a guide for chemists. It combines Volumes 1–4 of the print version into an interactive database of spectra, which enables users to undertake spectra analogue searches, and assists with identifying unknown entities. It is particularly recommended for anyone involved in analysing a wide range of inorganic compounds and organic salts.

Advantages of the CD-ROM include the following.

- Reversible forms of spectra with regard to absorbance and transmittance.
- Visualization tools include multi-pane spectra display, pan, zoom and colour options.
- Manipulation tools for comparisons between spectra of known and unknown compounds.
- Spreadsheet wavenumber log creation—to overlay specimen spectra on known spectra.
- Spectra analogue search option via pattern recognition.
- Band assignment and selection capabilities.
- Provides vibration mode animations.
- Displays spectra based on user-supplied compound information.

A free demonstration version of the CD-ROM is available on application to Microinfo. This contains fewer spectra, but provides all the functional aspects of the full version.

The *Electronic Handbook of Infrared and Raman Spectra of Inorganic Compounds and Organic Salts* is available from Microinfo, for the one-time purchase price, before 31 January 1999, of £1830.00 plus £10.00 UK postage and packing, plus VAT.

To view recent press releases, visit the Microinfo web site at: http://www.microinfo.co.uk/

### Database of palladium chemistry: reactions, catalytic cycles and chemical parameters on cd-rom

The *Database of Palladium Chemistry* on CD-ROM, Version 1.1, includes reactions, catalytic cycles and chemical parameters. It is an update of the previous version. The new edition contains 3850 reactions, 87 types of mechanisms and now includes papers using combinatorial chemistry.

The CD-ROM incorporates a search engine enabling users to find reagents, reaction products, or the underlying mechanisms. For each type of mechanism, searching is possible by author name, periodic structure, substructure, solvent or catalyst ligand.

Additional features on this CD-ROM include the following.

- Chemical parameters related to reactions and types of mechanism.
- Searchable via reagents, reaction products and the underlying mechanisms.
- Three hundred and fifty new reactions and two new types of mechanisms.
- Combines 10 years of literature into a single searchable resource.

The *Database of Palladium Chemistry* is available from Microinfo, for the price of £650.00 plus £5.00 UK postage and packing, plus VAT.

A free copy of the *Microinfo Electronic Media Directory*, containing details of over 3000 CD-ROM, Internet and diskette titles, is available on request.

N.B. Prices are subject to change without notice. For further information, please contact Brian Bridge at Microinfo, e-mail: brian.bridge@microinfo.co.uk To view recent press releases, visit the Microinfo web site at: http://www.microinfo.co.uk/

### Ninety-six- or 384-well microplate pipetting workstation

The RapidPlate Workstation is now available for pipetting using 384-well plates. The workstation improves throughout and eliminates well-to-well variability by delivering reagent to 96 wells of a microplate simultaneously. You can pipette to and from both 96- and 384-well plates. The pipettor aspirates and dispenses in 1-μl increments, and can pipette volumes to and from 1 μl–200 μl from reagent reservoirs, deepwells and microplates. The six-position turntable can accommodate plates, tips and reservoirs, and an on-line filling or tip-washing option is available.

The Windows™ software allows adjustments and optimization of pipetting variables for procedures including plate replications, reagent addition, multi-aspirate and serial dilutions. The flexible, stand-alone workstation can also be integrated into a Virtuoso™ or Allegro™ robotic system.

Zymark Corporation, headquartered in Hopkinton, MA, is a worldwide leader in laboratory automation for pharmaceutical and biotechnology applications. Zymark serves the wordwide pharmaceutical and biotechnology industries employing about 300 people in the USA, Canada and Europe, and with authorized sales and service distributors throughout the rest of the world.

*For more information contact Sharon Correia. Tel: +1 508 497 6403, sharon.correia@zymark.com*

### New size exclusion/gel permeation chromatography software now available from justice laboratory software UK

Justice Laboratory Software UK has announced a full-featured size exclusion chromatography (SEC) software package that is fully integrated with Chrom Perfect™ for Windows. With three modes of operation, the software allows one to retrieve a chromatogram collected by Chrom Perfect and produce SEC plots and reports interactively. It also allows one to produce reports and plots interactively after each run or by batch reprocessing a series of runs. Specifications come from an SEC method file that the chemist prepares ahead of time.

The SEC/GPC software package supports a wide variety of calaborations, including: narrow standard; broad standard; and universal calibration. The software fits the calibration curve with a polynominal up to degree 5.

This software can transfer plots and reports to other software, e.g. EXCEL, Lotus 1-2-3 or most word processing programs. It leaves SEC results in ASCII file format for transfer. It can also leave the plot in a Windows® metafile or bitmap file for transfer to a word processor or desktop publishing software. SEC provides a wide variety of calibrations and reports, and produces accurate and reproducible measurements of molecular weight.

Justice Laboratory Software UK offers a highly comprehensive line of high-technology software products for the analytical laboratory. From NT-based client server chromatography systems, to single user basic data systems, Justice Laboratory Software UK meets the demanding needs of chromatography applications. Their products offer multi-vendor instrument interfacing and distributed data processing for total integration of the laboratory.

*For more information contact Jim Russell at +44 (0)1337 828494 or visit the website at http://www.JusticeUK.com*

### Atomic fluorescence gaining acceptance as standard method for low-level mercury analysis

In 1988, P S Analytical introduced the world's first fully automated mercury analyser based on atomic fluorescence. The technology was developed in conjunction with ALcontrol Laboratories and the methodology accepted by the UK Environment Agency's Standing Committee of Analysts as a suitable standard. In many countries, however, legislation and standard methods were predominantly based on atomic absorption.

Since this time, P S Analytical has been working with many standards groups around the world to incorporate the atomic fluorescence approach. In the USA, a new 1631 method has been published which proposes atomic fluorescence albeit using an amalgamation stage. Whilst such stages are prone to contamination and carry-over, the new range of P S Analytical Millennium Merlin systems can achieve the levels of detection required without recourse to a preconcentration step. In the USA, a method (7474) for sediment and tissue samples has been published using the atomic fluorescence approach. In Europe, the Community European Normalization TC230 working groups have formulated a method for water samples in the range 1–100 ppt based on atomic fluorescence. This is in the final stages of acceptance as an appropriate standard. In several countries in the European Community, national groups are accepting atomic fluorescence technology as being the more reliable easy-to-use option for low-level Hg analysis.

Recently, P S Analytical and Red Control SA, Avda. Blasco Ibanez 153, Bajos, Valencia, Spain, have worked together to generate an endorsed method suitable for use in the Spanish water industry. Red Control have reported reliable data for water, soils and sludges. The methodology which was originally developed on the Merlin Plus technology can be readily transferred to the Millennium range with the added advantages of improved precision and lower detection levels.

The Millennium Excalibur system extends the range of analytes to the hydride-forming elements, especially arsenic, antimony and selenium at ppt levels.

PS Analytical has also developed the Sir Galahad II instrument which is a market leader for the analysis of mercury in the National Gas Industry. It can be used in both on-line and off-line modes.

### References

*Methods for the Examination of Waters and Associated Materials*

*Mercury in Waters, Effluents, Soils and Sediments,* etc., *Additional Methods* (1985). Published by Her Majesty's Stationery Office, London, UK.

*For further information on these and other PS Analytical products, either visit our Website at http://www.psanalytical.demon.co.uk or contact us at PS Analytical Ltd, Arthur House, Crayfields Industrial Estate, Main Road, Orpington, Kent, BR5 3HP, UK. Tel (Int): +44 (0)1689 891211; Fax (Int): +44 (0)1689 896009; e-mail: psa@psanalytical.demon.co.uk*

### Shimadzu technique for food safety testing by analysis of plastic packaging

Printed plastic film—a common packaging material for many food products—contains solvent residues, e.g. alcohols, hydrocarbons and ketones which, because of their high volatility, can be easily absorbed by food and then by the people who eat it.

The main problem for food and packaging materials manufacturers has been the accurate quantification of components in packaging material so that leaching of solvent to foodstuffs can be minimized, and optimum hygeine and health and safety standards attained.

The headspace analysis method using a Shimadzu gas chromatograph CG-14A and HSS-2B headspace system is a particularly useful technique for this; its easy, user-friendly handling system and high level of automation make it a fast, accurate and efficient approach to the routine determination of the solvent content of packaging materials.

Quantification is possible via conventional external calibration, or by standard addition techniques where solutions of different concentrations are added to the sample for analysis.

In all tests using the Shimadzu headspace methodology and instruments, the approach has been found to yield consistently accurate and sensitive results in the qualitative and quantitative analysis of volatile solvents in printed plastic film, giving analysts a fast and reliable technique for greater confidence in food safety and hygiene.

*For more details on Shimadzu headspace technology—including free copies of scientific application notes describing the technique in use—and information on other Shimadzu products for foodstuff and drinks manufacturing and processing, contact Shimadzu on +44 (0)1908 552200 or e-mail Sales@Shimadzu.co.uk*

*For more information, please contact: Shimadzu UK, Mill court, Featherstone Road, Wolverton Mill South, Milton Keynes MK12 5RE UK; Tel.: +44 (0)1908 552200; Fax.: +44 (0)1908 552211; e-mail: Sales@Shimadzu.co.uk or Ross Heaven, Strong Words, 32 Cranstoun Street, Northampton NN1 3BH, UK; Tel/Fax: +44 (0)1604 250221; e-mail: rossheaven @aol.com*

### Announcement

### ISLAR '99—Keynote Talks by Novartis and Roche Biosciences Highlight the 17th International Symposium on Laboratory Automation and Robotics

ISLAR '99, the seventeenth in a continuing series of symposia dedicated to laboratory automation and robotics, will be held at the Boston Park Plaza Hotel during 17–20 October 1999. A strong emphasis will be placed on presentations by senior laboratory managers and on applications in drug discovery research. The preliminary programme details many of the presentations and session topics as well as short courses and interactive discussion groups. Visit the ISLAR Web pages for more information, including abstracts of the presentations (http://www.islar.com).

The opening plenary session will feature two keynote speakers. Stefanie G. Nair PhD, Head of Quality Assurance at Novartis Pharmaceuticals Corporation, will speak on 'Working smarter: automation as a strategy for laboratory efficiency objectives'. Roger L. Whiting PhD, Senior Vice President of Roche Bioscience, will discuss 'Frontiers in drug discovery: use of new technologies'.

Parallel podium presentations and afternoon poster sessions will cover a variety of automation topics in the following areas:

- drug discovery
- pharmaceutical development and quality control
- bioanalytical research
- chemical and related industries
- managing laboratory automation
- emerging technologies for combinatorial chemistry
- compound preparation and distribution
- high throughput structural characterization
- high throughput screening

- ultrahigh throughput screening and assay miniaturization
- automation technologies for genomics
- secondary screening
- assay optimization and methods development
- data management/data handling and bioinformatics
- introduction to laboratory robotics
- automation strategies for assay development and primary and secondary screening
- dissolution testing: theory, practice and automation techniques
- bioanalytical automation: trends, technology and new developments
- EasyLab® programming: tips and tricks
- PCS™: tips and tricks

The three-day programme consists of over 120 podium and poster presentations describing laboratory robotics and laboratory workstation procedures from pharmaceutical, biotechnology and chemical laboratories. The symposium's technical presentations and informal discussions provide an excellent opportunity for laboratory managers, automation users and novices to exchange ideas and applications experience.

*For more information write or call: Christine O'Neil, ISLAR, 68 Elm Street, Hopkinton, MA 01748, USA. Tel: +1 508 497 2224, Fax: +1 508 435 3439, e-mail: islar@islar.com, URL—http://www.islar.com*

### Meeting report

### ISLAR '98 Recognizes Pioneers in Laboratory Automation and Robotics

The 16th International Symposium on Laboratory Automation and Robotics, held at the Boston Park Plaza Hotel during 18–21 October, recognized six scientists for their roles in developing new applications and techniques, designing unique solutions to technical problems, and implementing laboratory automation in their organizations. Nominated by colleagues in their companies or industry, these scientists were selected for this recognition by a committee of previous award winners.

The six scientists included:

Muhammed Alburakeh, Barr Laboratories, Pomona, NY, USA;
Siegfried Brandtner, Grunenthal GmbH, Germany;
Harnath Doddapaneni, SmithKline Beecham Pharmaceuticals, King of Prussia, PA;
Jim Perry, Procter & Gamble, UK;
Richard Wildonger, Zeneca Pharmaceuticals, Wilmington, DE, USA;
Rudy Willebrords, Janssen Research Foundation, Belgium.

ISLAR '98 attracted about 700 scientists from 33 states and 19 countries for three days of presentations on laboratory automation and robotics. The premier technical symposium in this field featured a program with nearly 150 papers and posters, seven discussion groups and several workshops, and six hands-on short courses, including the following areas of interest.

- High throughput screening.
- Data management/data handling.
- Ultra high-throughput screening.
- Managing laboratory automation.
- Methods transfer/validation.
- Pharmaceutical analysis.
- Bioanalytical applications.
- Advanced topics.
- Automated synthesis.
- Compound purification, distribution and analysis in drug discovery.
- Managing pharmaceutical development and QC laboratories.
- Automated physical testing & chemical analysis.
- Consumer products/food applications.
- Predictive design/structural characterization.

*For more information contact: Christine O'Neil, ISLAR, 68 Elm Street, Hopkinton, MA 01748, USA; Tel.: +1 508/435-9500; Fax.: +1 508/435-3439; e-mail: islar@islar.com*

